# Supplementary material for: Characterization of cell states in biliary tract cancers identifies mechanisms of therapeutic resistance in a phase II trial of DKN-01/nivolumab
Source: medRxiv. 2024 Oct 8:2024.10.08.24315092. Preprint. [Version 1] doi: 10.1101/2024.10.08.24315092 (PMC11483019; doi:10.1101/2024.10.08.24315092)
Supplement: Supplement 1 — Figure S1. InferCNV profile of all tumor cells against normal cell references within the scSeq dataset. Figure S2. UMAP embedding of scRNAseq profiles of all cells obtained across biopsy samples labeled by (A) patient sample and (B) diagnosis histology. (C) Cell type proportions of all major cell types obtained by scRNAseq split by sample. Figure S3. (A) Table showing number and percentage of cells of each major cell subset that were DKK+. Scoring of (B) DKK1 ISH and (C) PD-L1 IHC staining on a subset of samples for which FFPE tissue was available. (D) Violin plots showing expression of DKK1, LRP6, a FZD gene signature and WNT gene signature split but major cell types. (E) Proportion of cells belonging to each major cell type split by cells that were DKK1+ and DKK1−. (F) Dot plot showing Wnt signature scoring across all major cell types split by pre- and on-treamtent. Figure S4. (A) UMAP embedding of all NK cells obtained by scRNAseq labeled by granular subsets. (B) Heatmap showing marker genes for NK cell subsets. Figure S5. Dot plot showing CKAP4 expression in all (A) major cell types, (B) CD4+ T cell subsets, (C) CD8+ T cell subsets, (D) myeloid subsets, and (E) NK cell subsets. Figure S6. Dot plot showing WNT signature scoring stratified by pre- and on-treatment samples in (A) CD4+ T cell subsets, (B) CD8+ T cell subsets, (C) myeloid subsets, and (D) NK cell subsets. Figure S7. (A) Gene programs identified by consensus non-negative matrix factorization (cNMF) on all T cells. Shown is a dot plot of the usage of each identified gene program within each myeloid subset. (B) Correlation heatmap of cNMF usages with known, annotated gene programs from an independent dataset (Pelka et al). (C) Changes over treatment timepoints in Usage 2 (GzmK), Usage 4 (proliferation), Usage 6 (proliferation) and Usage 8 (CXCL13) in CD4+ and CD8+ T cell subsets. Figure S8. (A)-(B) Gene programs identified by cNMF on all myeloid cells. Shown is a dot plot of the usage of each identified [file media-1.pdf]

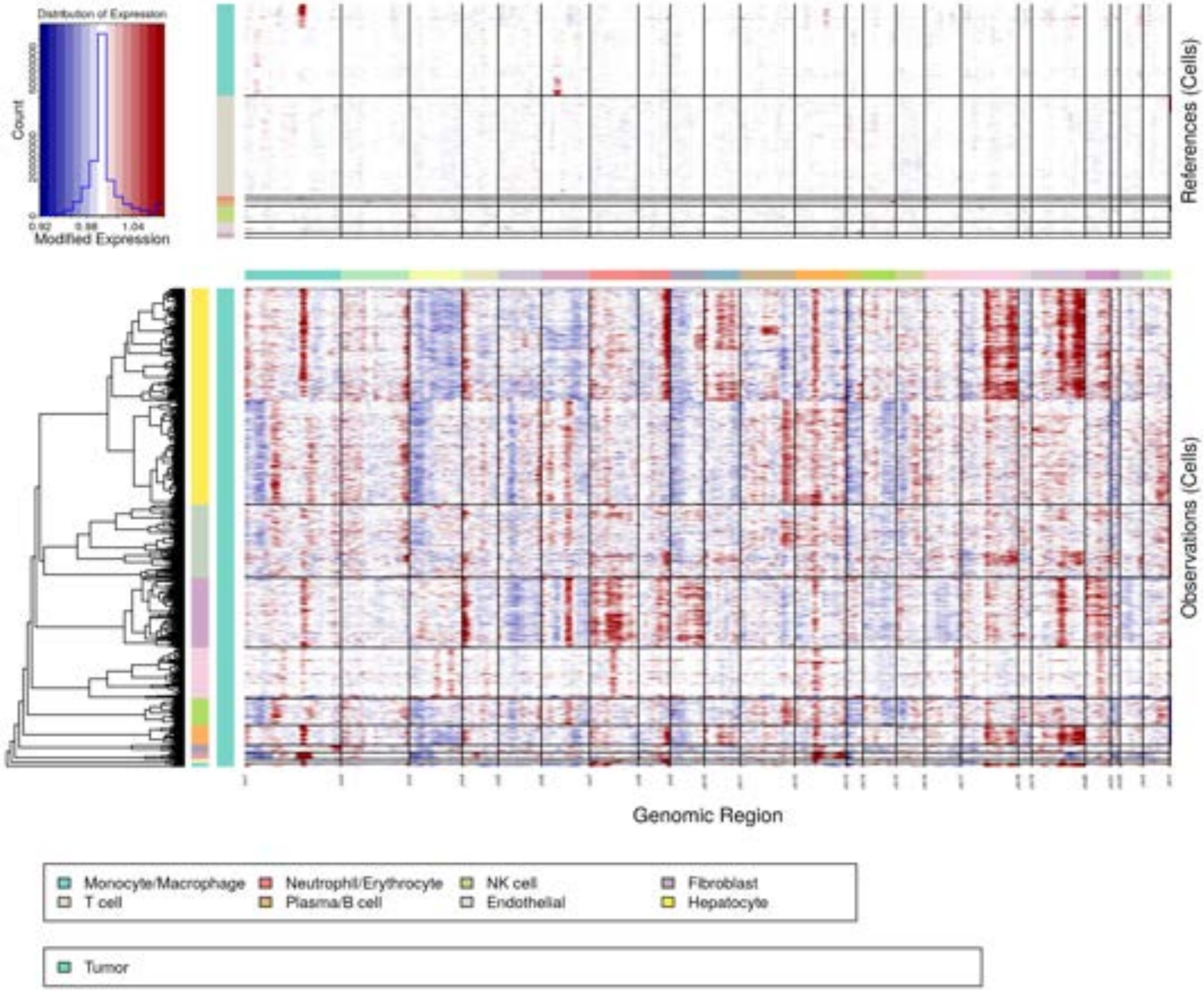

Distribution of Expression
Count
Modified Expression
References (Cells)
Observations (Cells)
Genomic Region
Monocyte/Macrophage
T cell
Neutrophil/Erythrocyte
Plasma/B cell
NK cell
Endothelial
Fibroblast
Hepatocyte
Tumor

Figure S1

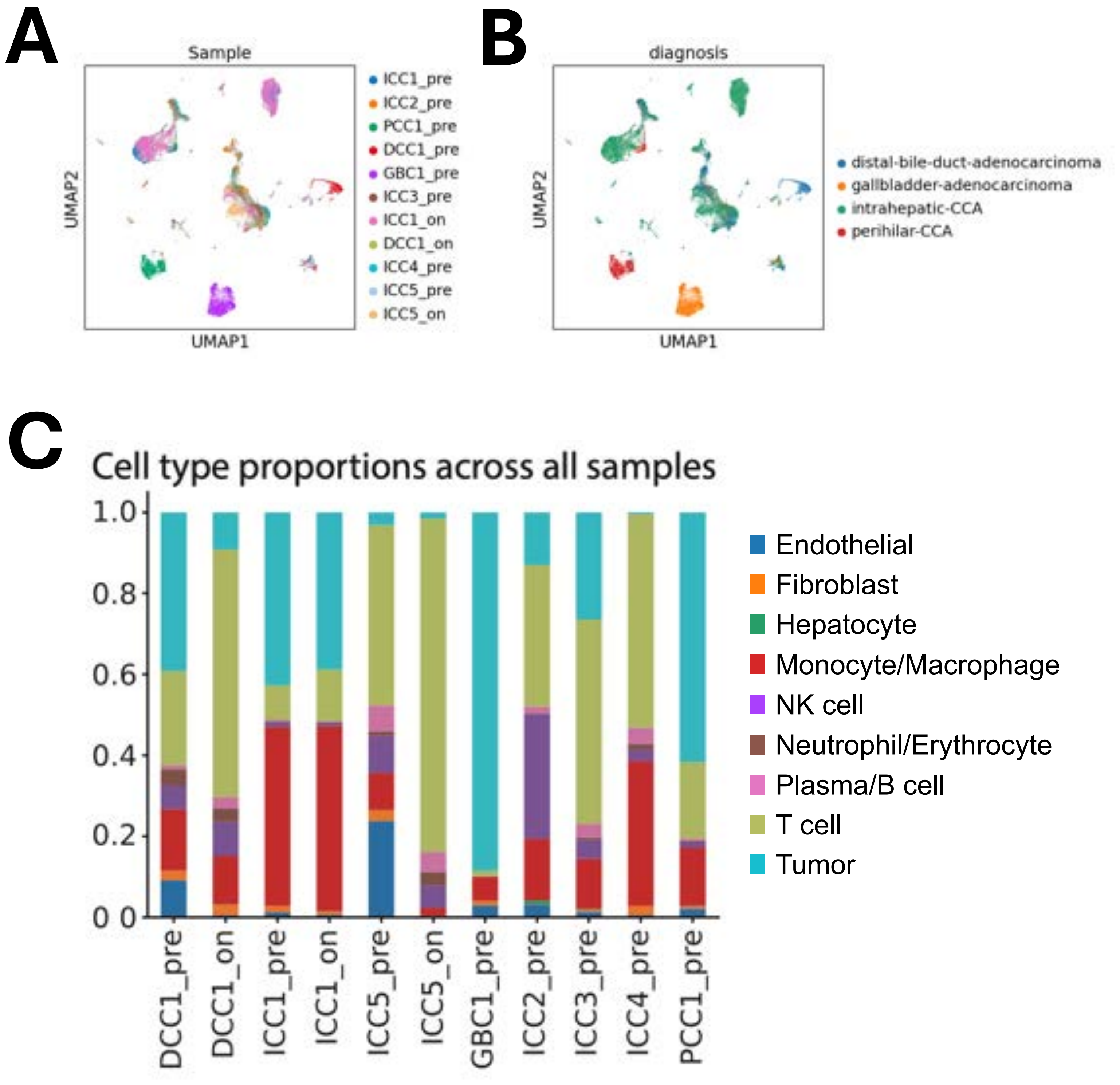
A
Sample
ICC1_pre
ICC2_pre
PCC1_pre
DCC1_pre
GBC1_pre
ICC3_pre
ICC1_on
DCC1_on
ICC4_pre
ICC5_pre
ICC5_on
UMAP1
UMAP2
B
diagnosis
distal-bile-duct-adenocarcinoma
gallbladder-adenocarcinoma
intrahepatic-CCA
perihilar-CCA
UMAP1
UMAP2
C
Cell type proportions across all samples
1.0
0.8
0.6
0.4
0.2
0.0
DCC1_pre
DCC1_on
ICC1_pre
ICC1_on
ICC5_pre
ICC5_on
GBC1_pre
ICC2_pre
ICC3_pre
ICC4_pre
PCC1_pre
Endothelial
Fibroblast
Hepatocyte
Monocyte/Macrophage
NK cell
Neutrophil/Erythrocyte
Plasma/B cell
T cell
Tumor

Figure S2

A

Cluster	DKK1-	DKK1+	% positive
Tumor	18,299	811	4%
T cell	12,952	9	< 0.1%
Mono/Mac	11,827	50	0.4%
NK cell	2,056	1	< 0.1%
Endothelial	1,460	1	< 0.1%
Plasma/B	729	0	< 0.1%
Fibroblast	616	10	1.6%
Neutrophil/erythrocyte	514	1	0.2%
Hepatocyte	44	0	< 0.1%

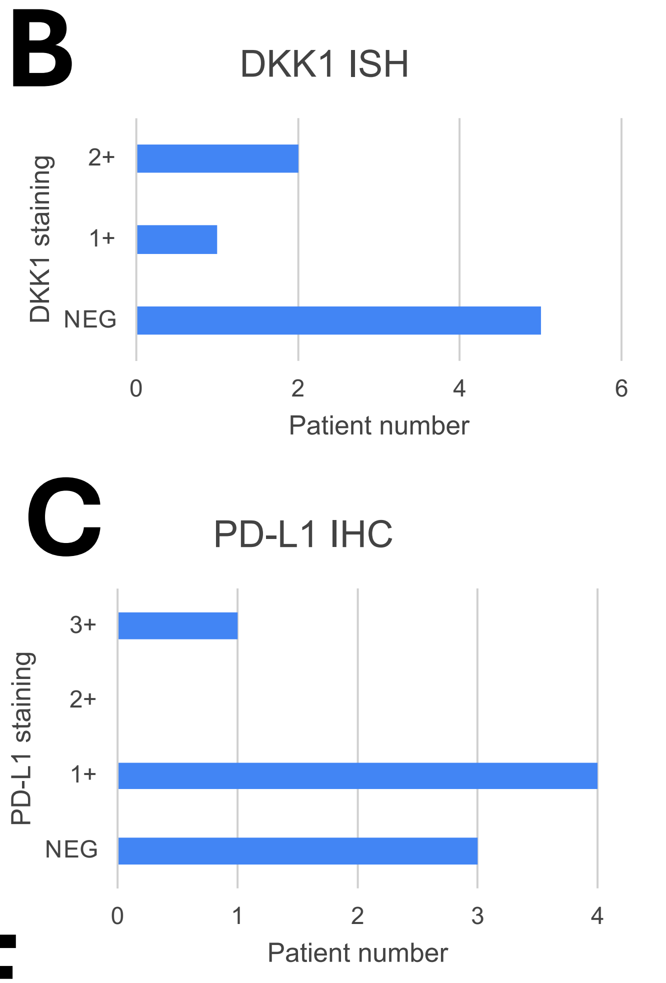

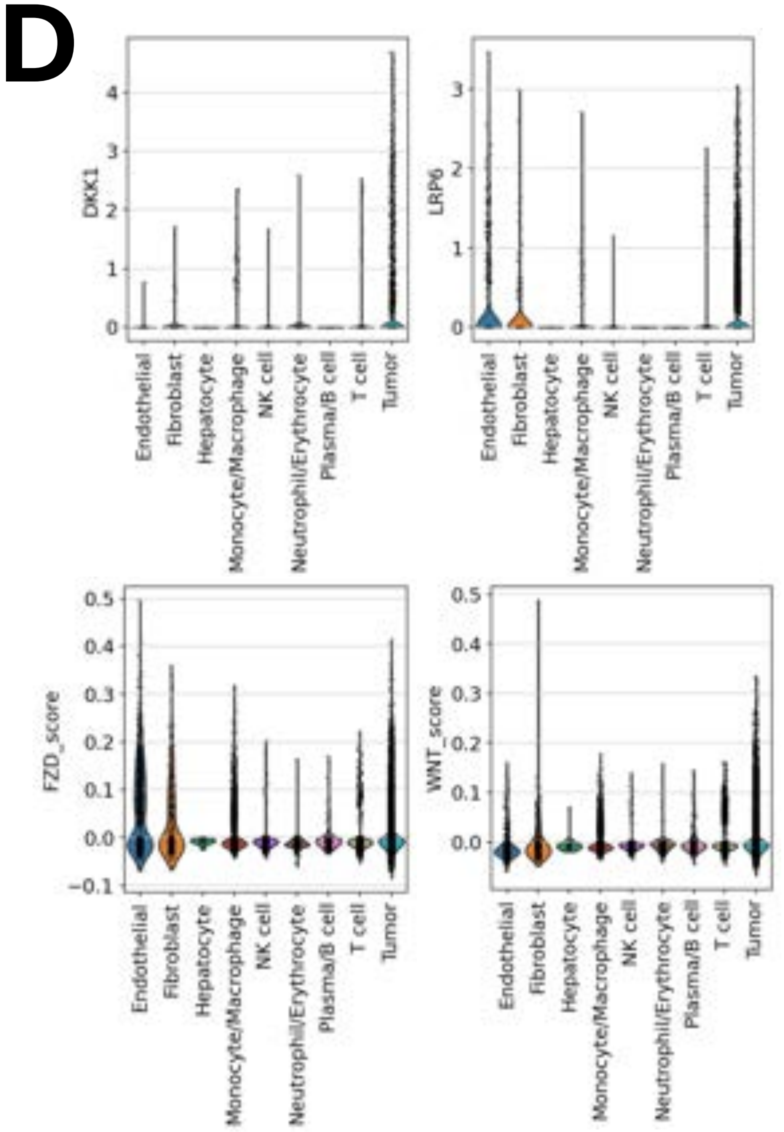

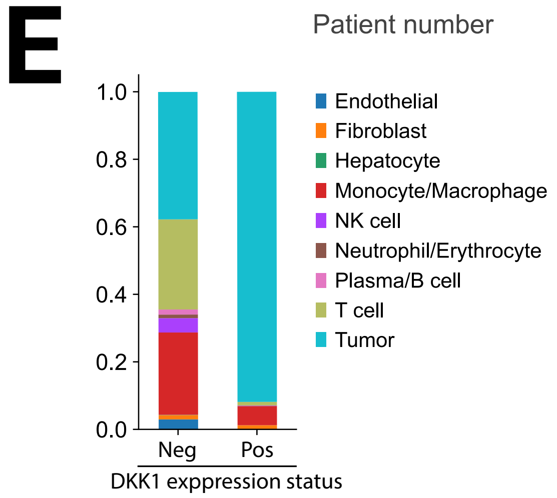

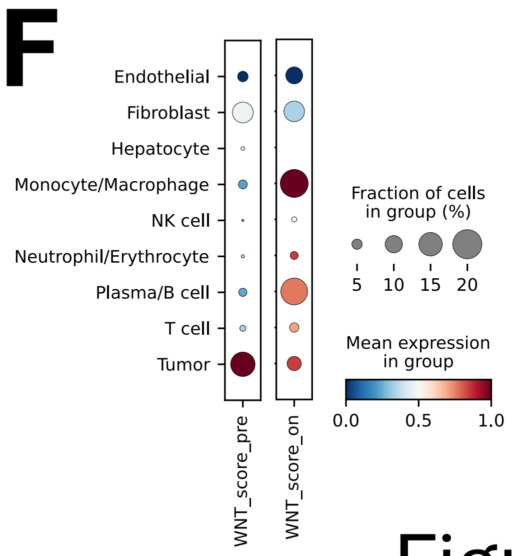

Figure S3

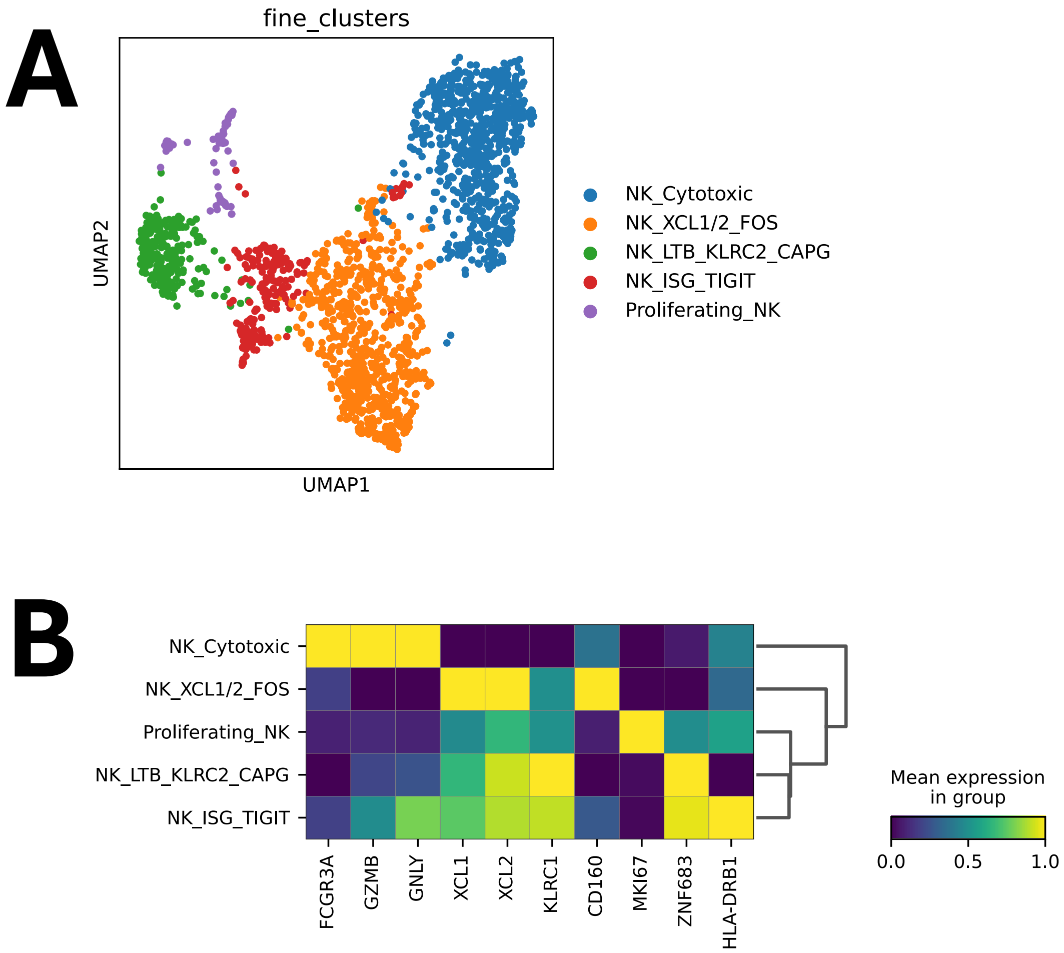
A
fine_clusters
UMAP2
UMAP1
NK_Cytotoxic
NK_XCL1/2_FOS
NK_LTB_KLRC2_CAPG
NK_ISG_TIGIT
Proliferating_NK
B
NK_Cytotoxic
NK_XCL1/2_FOS
Proliferating_NK
NK_LTB_KLRC2_CAPG
NK_ISG_TIGIT
FCGR3A
GZMB
GNLY
XCL1
XCL2
KLRC1
CD160
MKI67
ZNF683
HLA-DRB1
Mean expression
in group
0.0
0.5
1.0

Figure S4

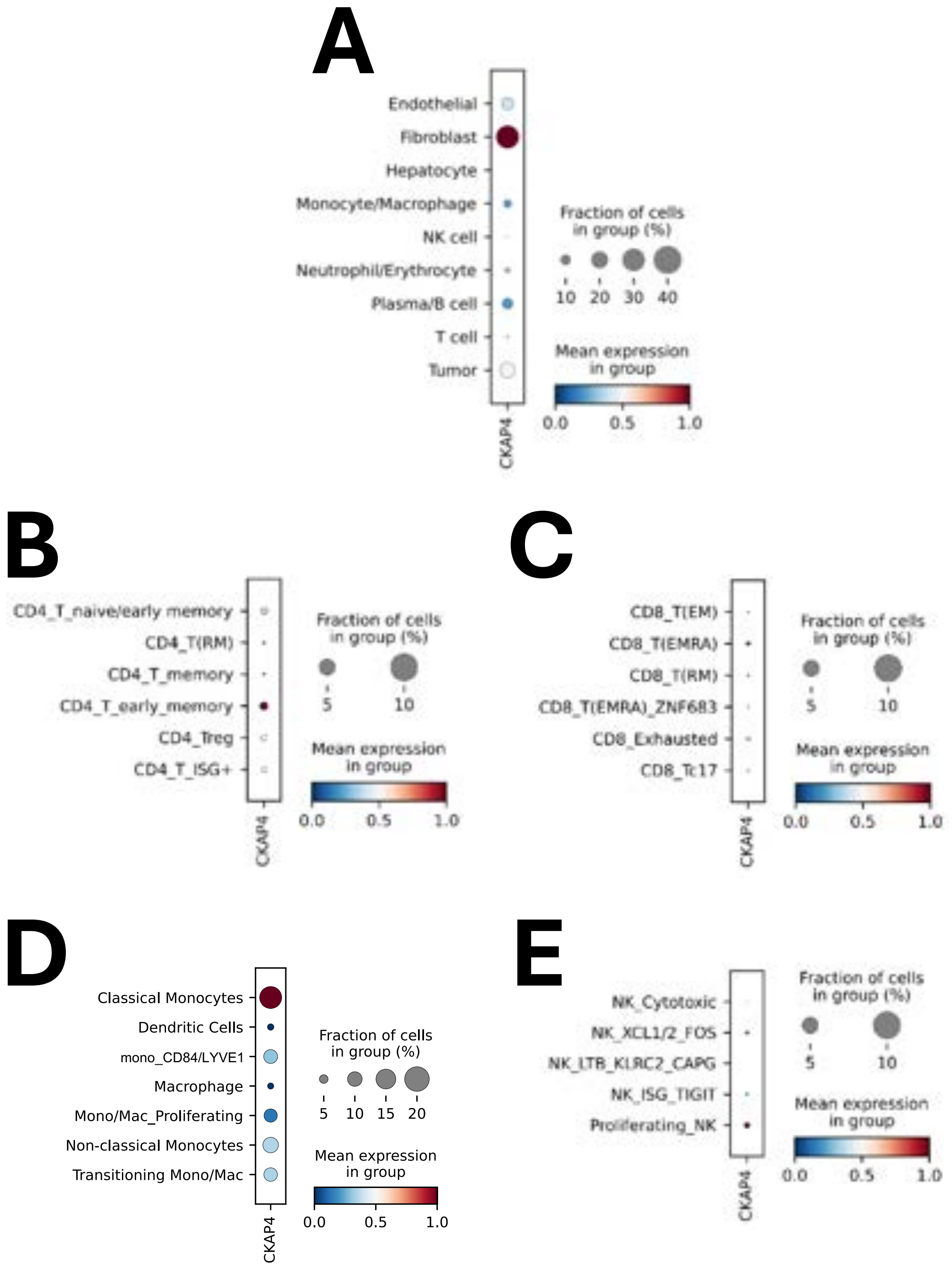
A
Endothelial
Fibroblast
Hepatocyte
Monocyte/Macrophage
NK cell
Neutrophil/Erythrocyte
Plasma/B cell
T cell
Tumor
CKAP4
Fraction of cells in group (%)
10 20 30 40
Mean expression in group
0.0 0.5 1.0
B
CD4_T_naive/early memory
CD4_T(RM)
CD4_T_memory
CD4_T_early_memory
CD4_Treg
CD4_T_ISG+
CKAP4
Fraction of cells in group (%)
5 10
Mean expression in group
0.0 0.5 1.0
C
CD8_T(EM)
CD8_T(EMRA)
CD8_T(RM)
CD8_T(EMRA)_ZNF683
CD8_Exhausted
CD8_Tc17
CKAP4
Fraction of cells in group (%)
5 10
Mean expression in group
0.0 0.5 1.0
D
Classical Monocytes
Dendritic Cells
mono_CD84/LYVE1
Macrophage
Mono/Mac_Proliferating
Non-classical Monocytes
Transitioning Mono/Mac
CKAP4
Fraction of cells in group (%)
5 10 15 20
Mean expression in group
0.0 0.5 1.0
E
NK_Cytotoxic
NK_XCL1/2_FOS
NK_LTB_KLRC2_CAPG
NK_ISG_TIGIT
Proliferating_NK
CKAP4
Fraction of cells in group (%)
5 10
Mean expression in group
0.0 0.5 1.0

Figure S5

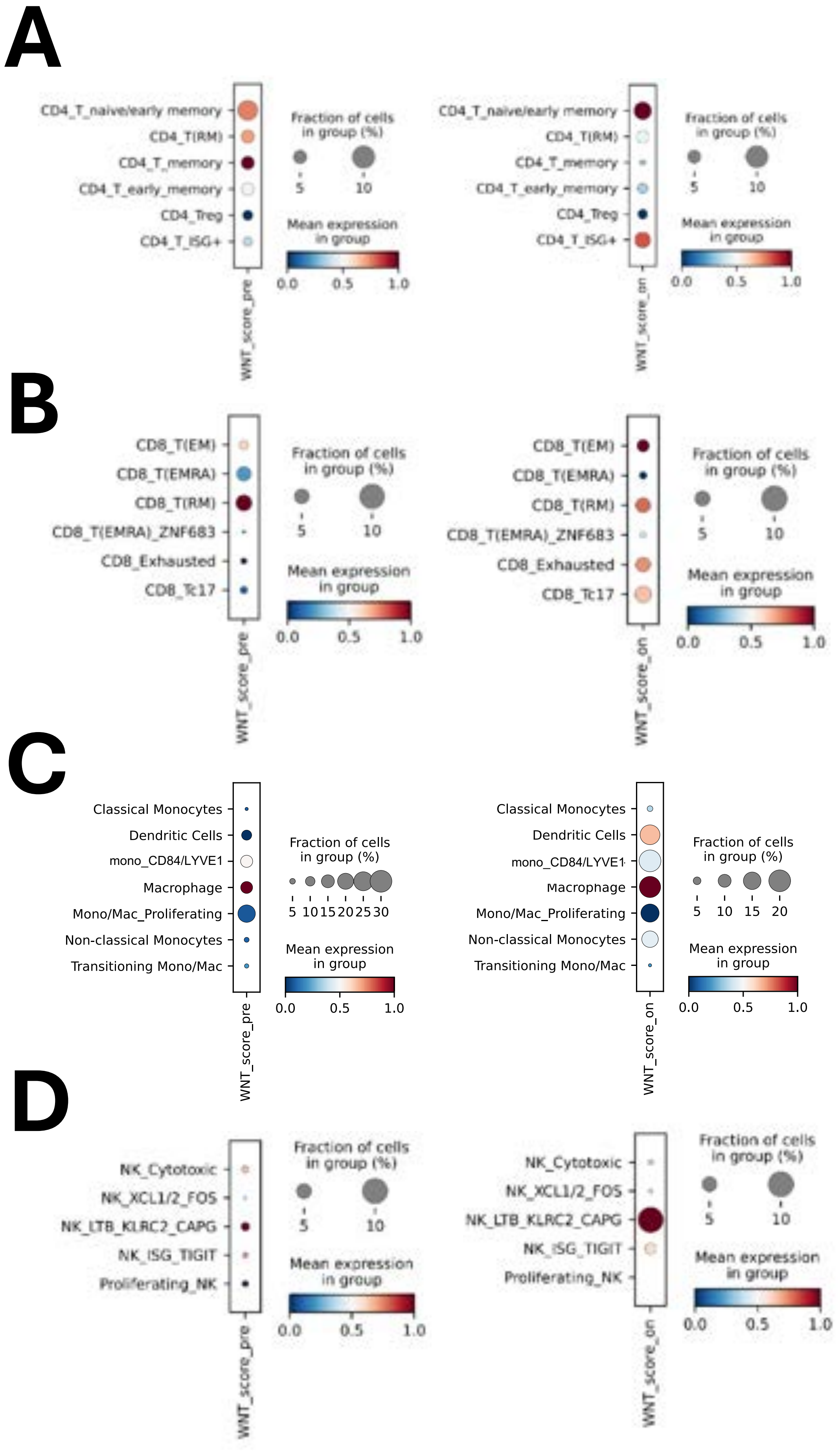
A
CD4_T_naive/early memory
CD4_T(RM)
CD4_T_memory
CD4_T_early_memory
CD4_Treg
CD4_T_ISG+
WNT_score_pre
WNT_score_on
Fraction of cells in group (%)
5
10
Mean expression in group
0.0
0.5
1.0
B
CD8_T(EM)
CD8_T(EMRA)
CD8_T(RM)
CD8_T(EMRA)_ZNF683
CD8_Exhausted
CD8_Tc17
C
Classical Monocytes
Dendritic Cells
mono_CD84/LYVE1
Macrophage
Mono/Mac_Proliferating
Non-classical Monocytes
Transitioning Mono/Mac
5 10 15 20 25 30
5 10 15 20
D
NK_Cytotoxic
NK_XCL1/2_FOS
NK_LTB_KLRC2_CAPG
NK_ISG_TIGIT
Proliferating_NK

Figure S6

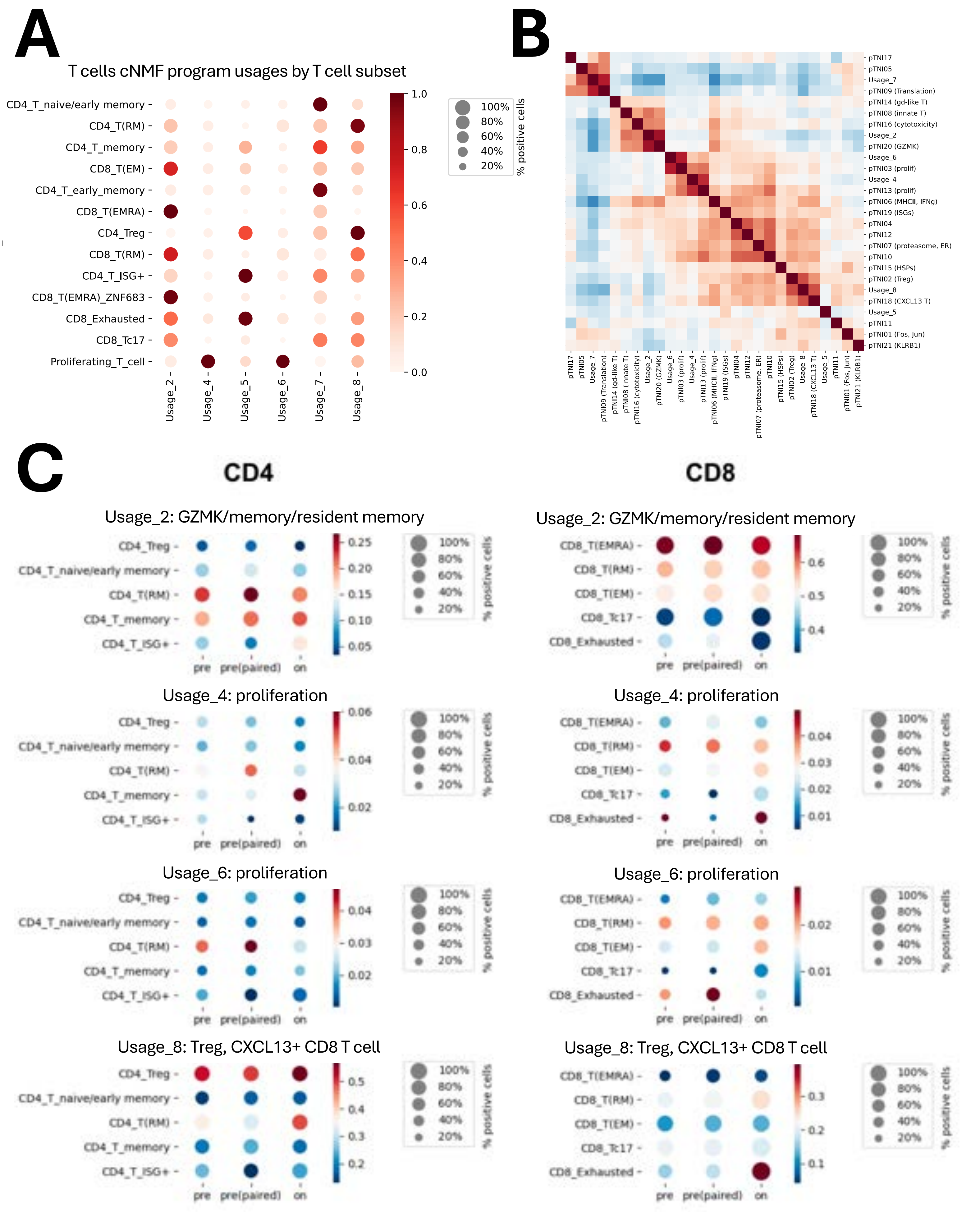
A
T cells cNMF program usages by T cell subset
CD4_T_naive/early memory
CD4_T(RM)
CD4_T_memory
CD8_T(EM)
CD4_T_early_memory
CD8_T(EMRA)
CD4_Treg
CD8_T(RM)
CD4_T_ISG+
CD8_T(EMRA)_ZNF683
CD8_Exhausted
CD8_Tc17
Proliferating_T_cell
Usage_2
Usage_4
Usage_5
Usage_6
Usage_7
Usage_8
1.0
0.8
0.6
0.4
0.2
0.0
100%
80%
60%
40%
20%
% positive cells
B
pTNI17
pTNI05
Usage_7
pTNI09 (Translation)
pTNI14 (gd-like T)
pTNI08 (innate T)
pTNI16 (cytotoxicity)
Usage_2
pTNI20 (GZMK)
Usage_6
pTNI03 (prolif)
Usage_4
pTNI13 (prolif)
pTNI06 (MHCII, IFNg)
pTNI19 (ISGs)
pTNI04
pTNI12
pTNI07 (proteasome, ER)
pTNI10
pTNI15 (HSPs)
pTNI02 (Treg)
Usage_8
pTNI18 (CXCL13 T)
Usage_5
pTNI11
pTNI01 (Fos, Jun)
pTNI21 (KLRB1)
C
CD4
CD8
Usage_2: GZMK/memory/resident memory
Usage_4: proliferation
Usage_6: proliferation
Usage_8: Treg, CXCL13+ CD8 T cell
CD4_Treg
CD4_T_naive/early memory
CD4_T(RM)
CD4_T_memory
CD4_T_ISG+
CD8_T(EMRA)
CD8_T(RM)
CD8_T(EM)
CD8_Tc17
CD8_Exhausted
pre
pre(paired)
on
% positive cells

Figure S7

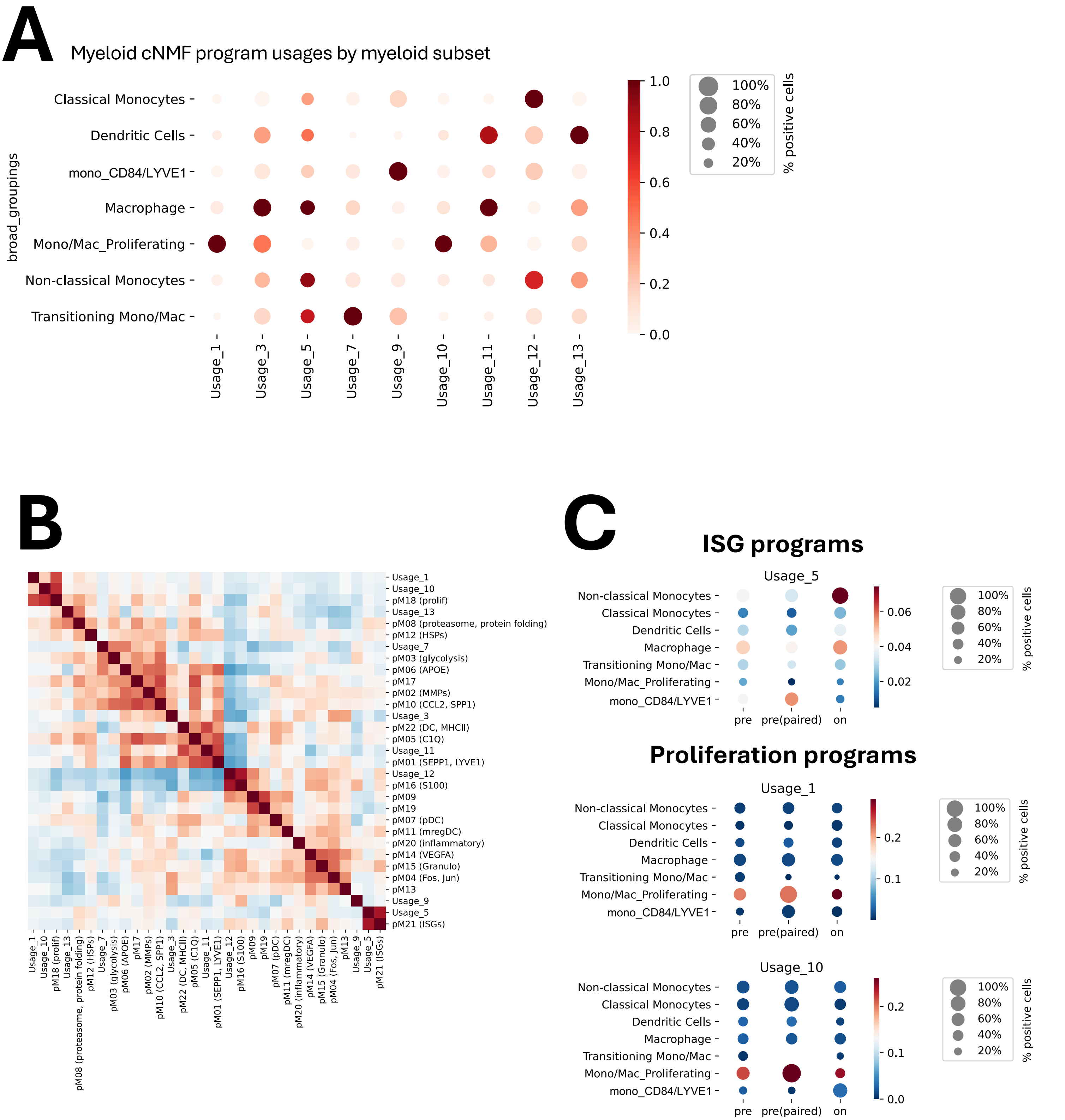

A
Myeloid cNMF program usages by myeloid subset
broad_groupings
Classical Monocytes
Dendritic Cells
mono_CD84/LYVE1
Macrophage
Mono/Mac_Proliferating
Non-classical Monocytes
Transitioning Mono/Mac
Usage_1
Usage_3
Usage_5
Usage_7
Usage_9
Usage_10
Usage_11
Usage_12
Usage_13
1.0
0.8
0.6
0.4
0.2
0.0
100%
80%
60%
40%
20%
% positive cells
B
Usage_1
Usage_10
pM18 (prolif)
Usage_13
pM08 (proteasome, protein folding)
pM12 (HSPs)
Usage_7
pM03 (glycolysis)
pM06 (APOE)
pM17
pM02 (MMPs)
pM10 (CCL2, SPP1)
Usage_3
pM22 (DC, MHCII)
pM05 (C1Q)
Usage_11
pM01 (SEPP1, LYVE1)
Usage_12
pM16 (S100)
pM09
pM19
pM07 (pDC)
pM11 (mregDC)
pM20 (inflammatory)
pM14 (VEGFA)
pM15 (Granulo)
pM04 (Fos, Jun)
pM13
Usage_9
Usage_5
pM21 (ISGs)
C
ISG programs
Usage_5
Non-classical Monocytes
Classical Monocytes
Dendritic Cells
Macrophage
Transitioning Mono/Mac
Mono/Mac_Proliferating
mono_CD84/LYVE1
pre
pre(paired)
on
0.06
0.04
0.02
100%
80%
60%
40%
20%
% positive cells
Proliferation programs
Usage_1
0.2
0.1
Usage_10
0.2
0.1
0.0

Figure S8

A

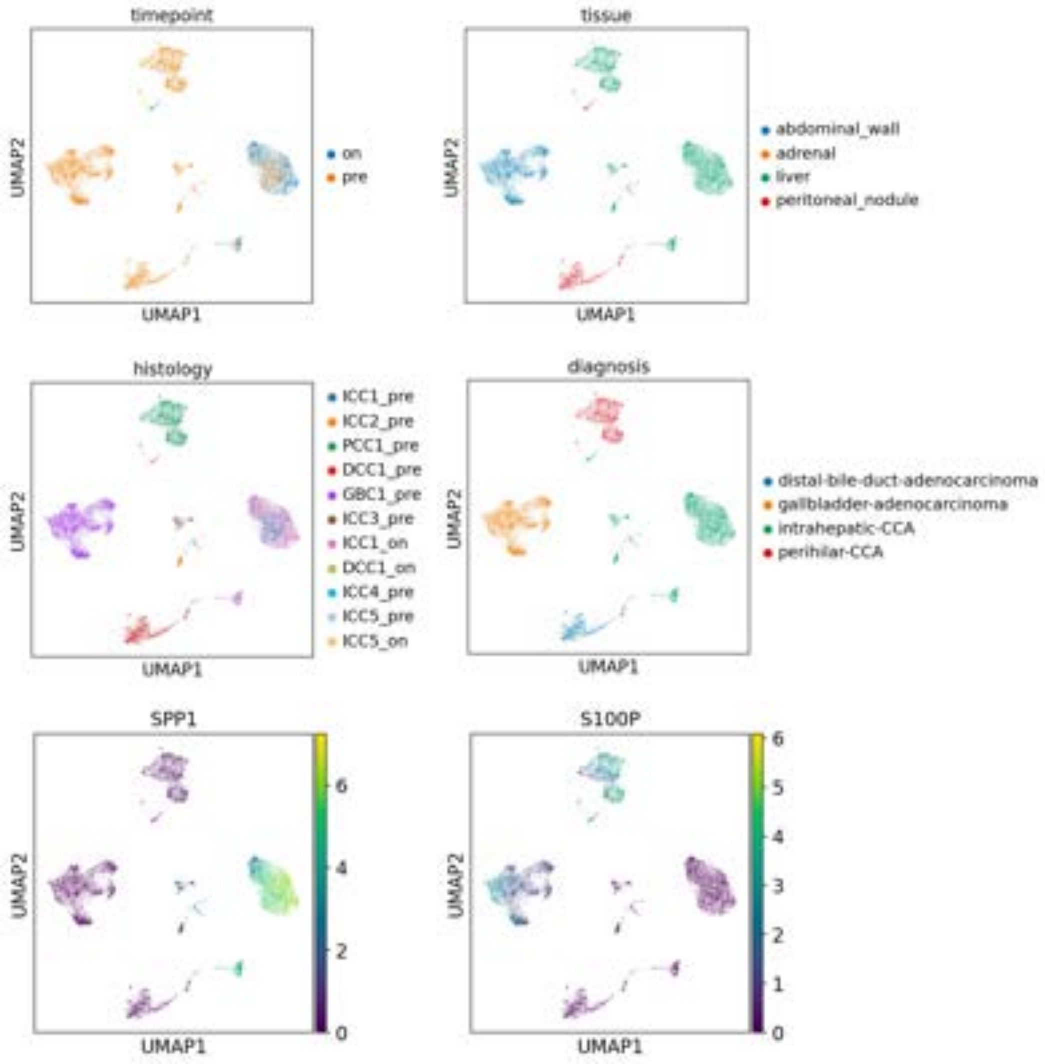
timepoint
tissue
histology
diagnosis
SPP1
S100P
UMAP1
UMAP2
on
pre
abdominal_wall
adrenal
liver
peritoneal_nodule
ICC1_pre
ICC2_pre
PCC1_pre
DCC1_pre
GBC1_pre
ICC3_pre
ICC1_on
DCC1_on
ICC4_pre
ICC5_pre
ICC5_on
distal-bile-duct-adenocarcinoma
gallbladder-adenocarcinoma
intrahepatic-CCA
perihilar-CCA

B

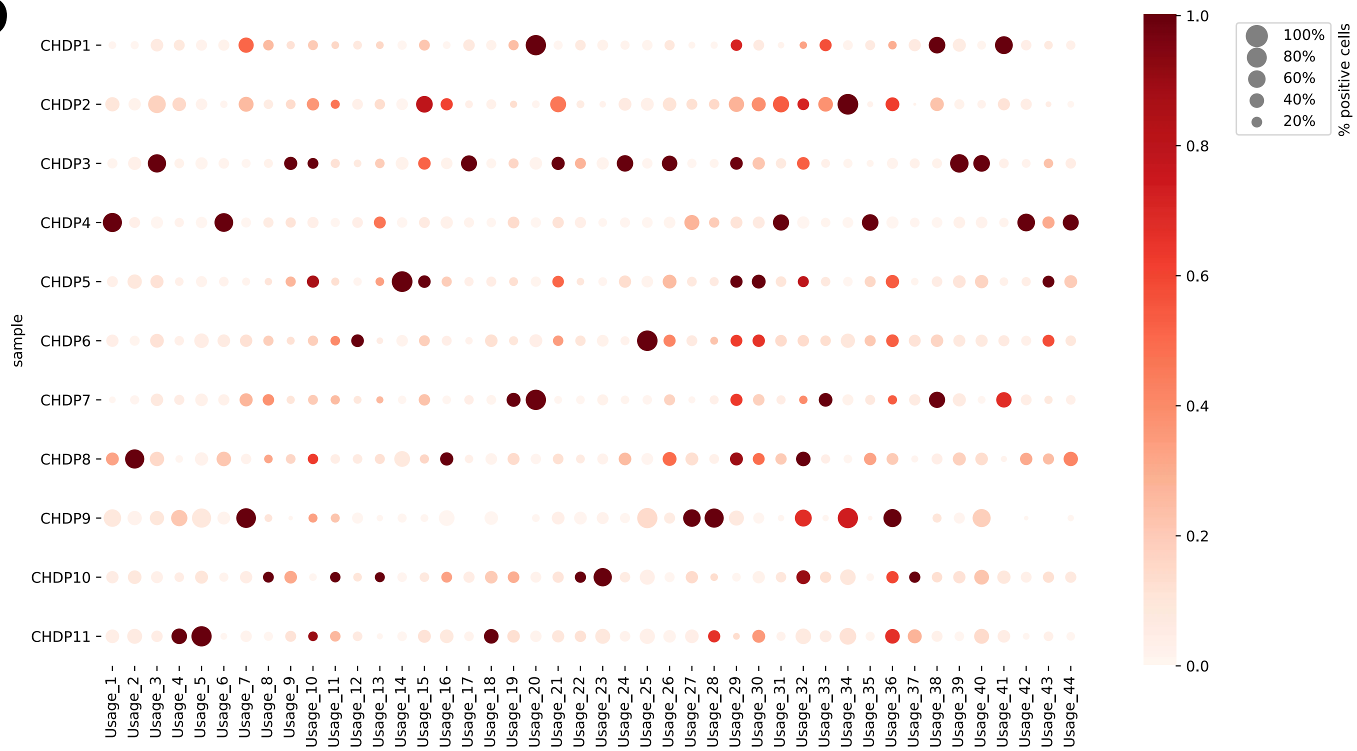
CHDP1
CHDP2
CHDP3
CHDP4
CHDP5
CHDP6
CHDP7
CHDP8
CHDP9
CHDP10
CHDP11
sample
Usage_1 … Usage_44
1.0
0.8
0.6
0.4
0.2
0.0
100%
80%
60%
40%
20%
% positive cells

Figure S9

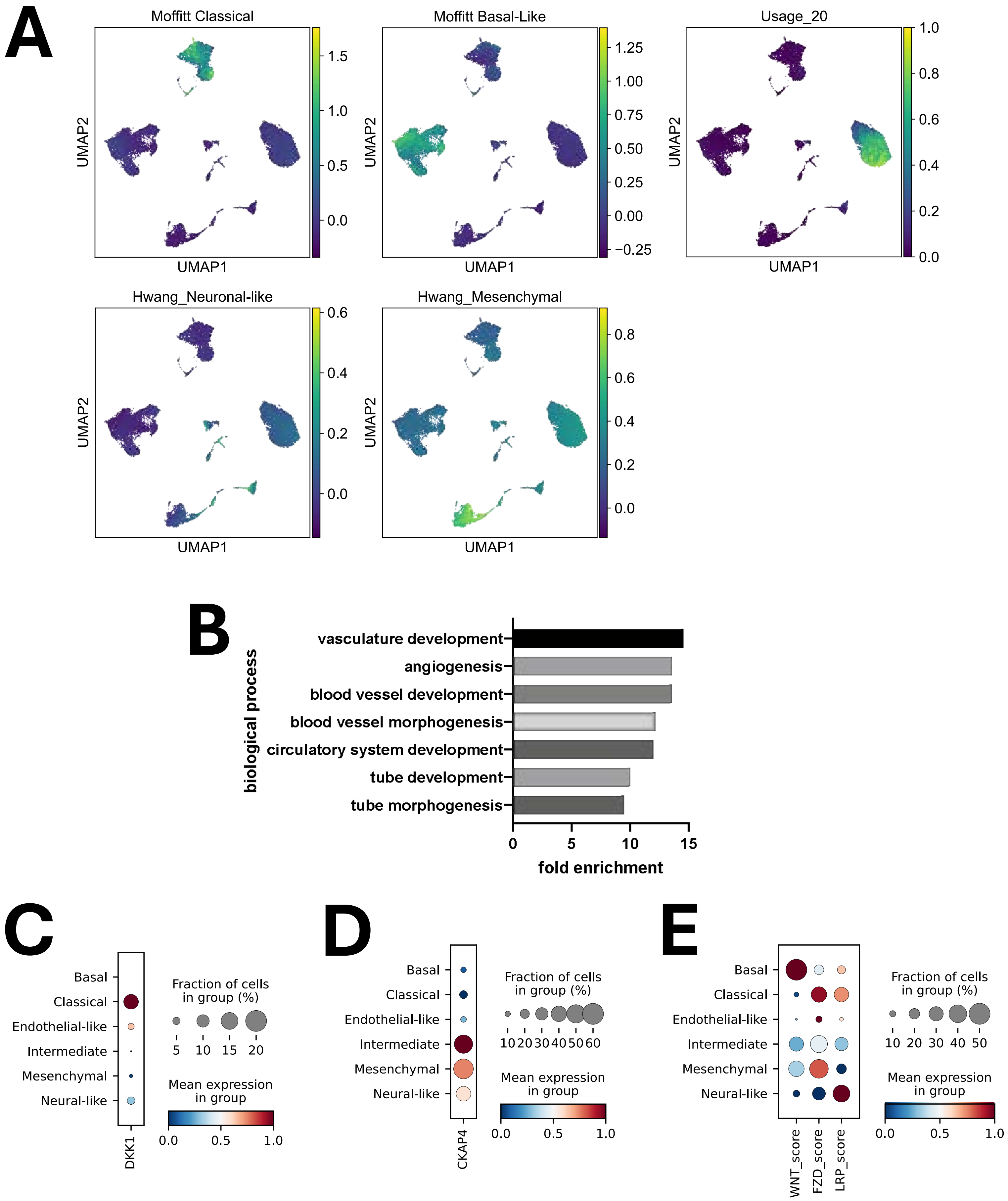
A
Moffitt Classical
Moffitt Basal-Like
Usage_20
Hwang_Neuronal-like
Hwang_Mesenchymal
UMAP1
UMAP2
B
vasculature development
angiogenesis
blood vessel development
blood vessel morphogenesis
circulatory system development
tube development
tube morphogenesis
biological process
fold enrichment
C
D
E
Basal
Classical
Endothelial-like
Intermediate
Mesenchymal
Neural-like
DKK1
CKAP4
WNT_score
FZD_score
LRP_score
Fraction of cells in group (%)
Mean expression in group

Figure S10

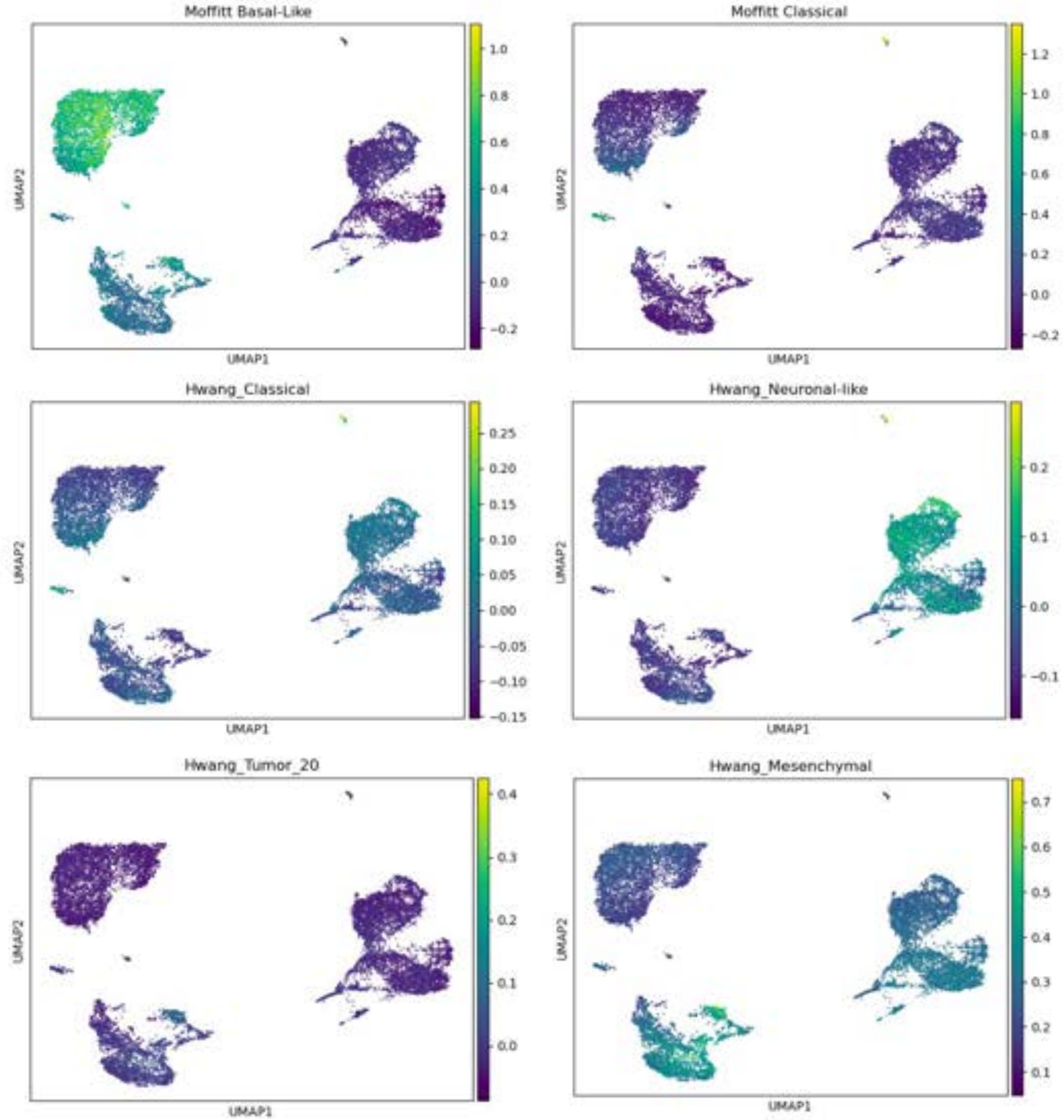
Moffitt Basal-Like
Moffitt Classical
Hwang_Classical
Hwang_Neuronal-like
Hwang_Tumor_20
Hwang_Mesenchymal
UMAP1
UMAP2

Figure S11

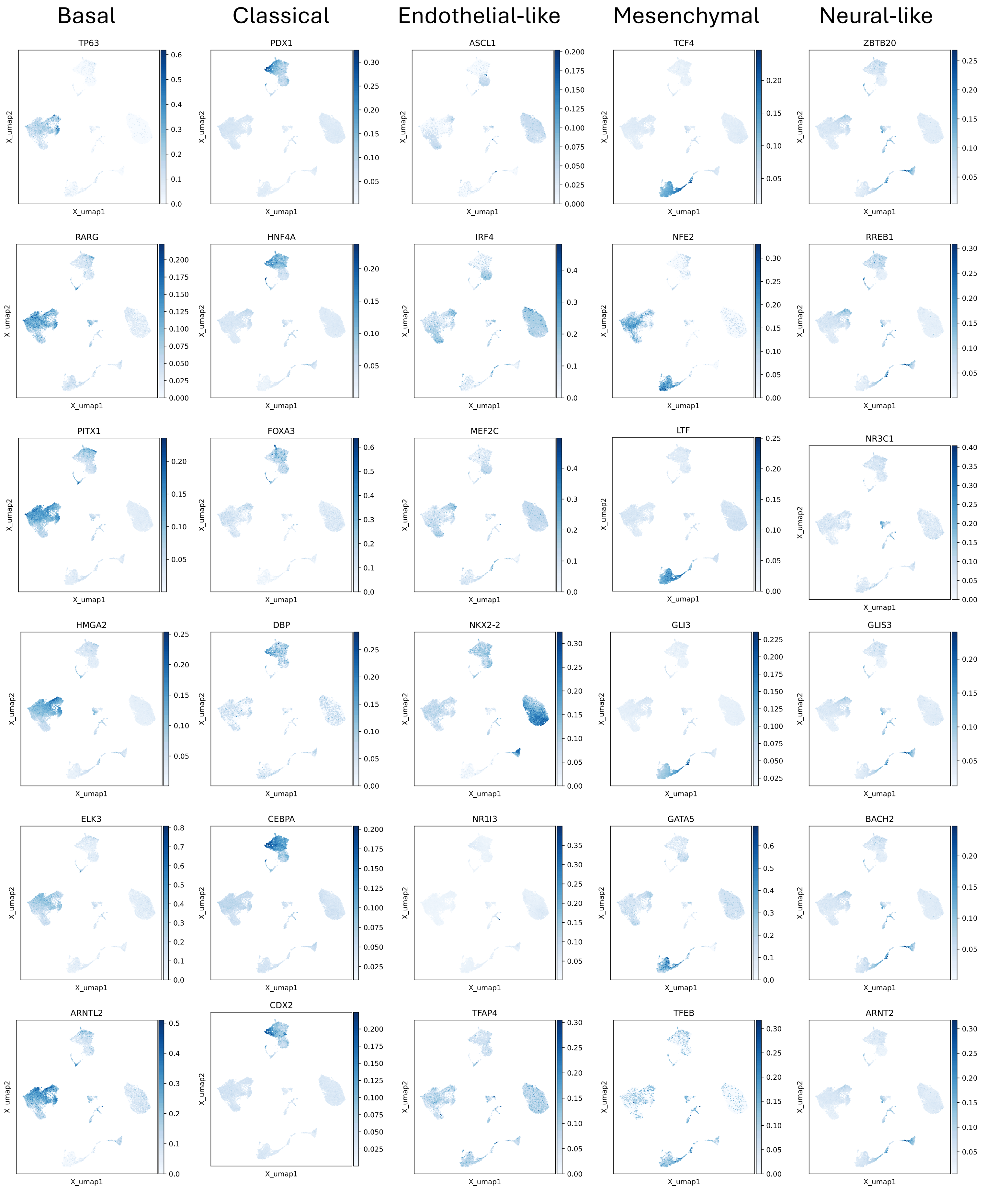
Basal
Classical
Endothelial-like
Mesenchymal
Neural-like
TP63
PDX1
ASCL1
TCF4
ZBTB20
RARG
HNF4A
IRF4
NFE2
RREB1
PITX1
FOXA3
MEF2C
LTF
NR3C1
HMGA2
DBP
NKX2-2
GLI3
GLIS3
ELK3
CEBPA
NR1I3
GATA5
BACH2
ARNTL2
CDX2
TFAP4
TFEB
ARNT2
X_umap1
X_umap2

Figure S12
